# The Ocular Surface Symptoms and Tear Film Parameters during and after COVID-19 Infection

**DOI:** 10.3390/jcm11226697

**Published:** 2022-11-12

**Authors:** Dominika Szkodny, Adam Wylęgała, Edyta Chlasta-Twardzik, Edward Wylęgała

**Affiliations:** 1 Chair and Clinical Department of Ophthalmology, Faculty of Medical Sciences, Zabrze Medical University of Silesia, 40-760 Katowice, Poland; 2 Department of Ophthalmology, District Railway Hospital, 40-760 Katowice, Poland

**Keywords:** COVID, tear film, dry eye disease, ocular surface, conjunctivitis

## Abstract

Purpose: This study aimed to evaluate the ocular surface parameters of post-COVID-19 patients when compared to healthy controls. Methods: Patients after symptomatic SARS-CoV-2 infection, as confirmed by a PCR test of their nasopharyngeal swab sample, were enrolled. Complete ophthalmic examination, including visual acuity test, intraocular pressure measurement (IOP), slit-lamp examination, tear osmolarity test, central corneal thickness, endothelial cell number measurements, non-invasive keratograph break-up time (NIKBUT), meniscus height, and the Schirmer’s test were performed and compared with the controls. Results: It must be noted that there were 36 COVID-19 patients and 25 control subjects included in the study. Eye itching and burning (19%) were the most common symptoms of COVID-19 infection, followed by a subjective decrease in vision (17%), conjunctivitis and eye pain were present in 11%, and 6% of the patients had episcleritis. The mean time from initial infection was 6.5 ± 3.9 (range 1–24 weeks). Meniscus height was not significantly changed between the COVID-19 (0.34 ± 0.13 mm) group and the control (0.33 ± 0.12 mm, *p* = 0.88) group. In addition, the NIKBUT-1 (*p* = 0.88; 7.22 ± 4.60 s and 6.91 ± 4.45 s) and NIKBUT average (*p* = 0.91, 12.30 ± 5.86 s and 11.77 ± 4.97 s) test results showed no significant change either. Neither was a significant result found in the IOP (*p* = 0.17, 14.56 ± 2.10 mmHg and 14.11 ± 1.96 mmHg); the Schirmer test (*p* = 0.18, 20.22 ± 7.92 mm and 20.02 ± 7.17 mm); Tosm (*p* = 0.16, 294.42 ± 54.51 mOsm/dL and 299.33 ± 5.65 mOsm/dL); CCT (*p* = 0.06, 549.15 ± 28.98 vs. 539.21 vs. 29.08 µm); nor the endothelial cell density (*p* = 0.07, 2516.64 ± 287.61 vs. 2454.21 ± 498.60 cells/mm^2^). Conclusions: Through this study it was not revealed that there were any significant differences between the post-COVID group and control group in the objective measurements of ocular surface conditions, when performed after the acute phase of COVID-19. The exact incidence and mechanism of ocular findings, especially dry eye disease, in correlation with SARS-CoV-2 requires further research.

## 1. Introduction

From the end of 2019, when a novel coronavirus (COVID-19) emerged from China, until the present, the world is still struggling with the effects of this new, infectious, coronavirus disease. The interest of scientists in this newly discovered pathogen has resulted in numerous symptoms—regarding different organs— to be described in the course of COVID-19 disease infecting populations on the global stage. Though the retinal and choroidal symptoms have been reported mainly as a casuistry, the ocular surface has been also investigated as a potential infection zone [1,2]. The most common ophthalmic manifestation of COVID-19 is conjunctivitis [3]; however, it is known to be a rare symptom as the disease affects mainly the respiratory tract. Moreover, SARS-CoV-2 RNA has been detected, via using a reverse transcriptase polymerase chain reaction, in the tears and conjunctival secretions of COVID-19 patients with conjunctivitis [4]. Furthermore, Xie et al. also reported two cases with strong positive testing results of SARS-CoV-2 without conjunctivitis. Apart from conjunctivitis, the ocular manifestations of SARS-CoV-2 include hyperemia, ocular irritation, foreign body sensation, epiphora, and chemosis [5]. The prevalence of ocular symptoms varies between studies, ranging from 0.8% to 31.6% [6]. The complications following coronavirus infection, after the acute phase, have been observed. the World Health Organization created a definition of a “long–COVID-19” condition depending on certain symptoms or impairments, present at least 3 months after the onset of infection and that was unable to be explained by another diagnosis [7]. The most commonly reported symptoms included fatigue, shortness of breath, muscle pain, joint pain, headache, cough, chest pain, altered smell, altered taste, and diarrhea [8]. However, the knowledge about the impact of post-COVID-19 on the ocular surface is also limited. Some findings in patients examined a month after hospitalization has been found, such as a greater ocular surface disease index (OSDI) score, lower Schirmer’s test results, and shorter tear break-up times (TBUT), when compared to the control group [6]. Nevertheless, more research is required to confirm these findings.

As such, the purpose of this study was to evaluate the ocular surface parameters of patients after COVID-19 when compared to healthy controls.

## 2. Materials and Methods

This single-center, cross-sectional study was conducted from December 2020 until March 2021, followed the tenets and guidelines of the Helsinki Declaration, and was approved by the bioethical committee of the Silesian Medical University. Adult patients after symptomatic SARS-CoV-2 infection, when confirmed by a PCR test of a nasopharyngeal swab sample, at least one week after recovery were enrolled. Patients unable to take part in the study due to their general condition, whether they were underage, were with asymptomatic COVID-19 disease, or without confirmation of the coronavirus infection by PCR test were excluded. The control group consisted of healthy students and employees of the Railway Hospital in Katowice without a history of past COVID-19 and after a negative PCR test result was confirmed. Detailed medical histories regarding ophthalmic, systemic diseases, and ocular symptoms during SARS-CoV-2 were collected from each patient. Complete ophthalmic examination, including visual acuity test, intraocular pressure measurement (IOP), slit-lamp examination, tear osmolarity test, central corneal thickness, endothelial cell number measurements, non-invasive keratograph break-up time (NIKBUT), meniscus height, and the Schirmer’s test were all performed. All ocular surface tests were measured at the same center and at the same time, i.e., between 1 and 3 pm, for both the study and control groups. Tear film osmolarity was assessed with the TearLab Osmolarity System (TearLab, Escondido, CA, USA) after calibration of the instrument. Further, NIKBUT and meniscus height were measured with Oculus Keratograph 4 (Oculus, Weltzar, Germany). All parameters were analyzed and compared between groups.

## 3. Statistical Analysis

The normality of the data was measured using the Shapiro–Wilk test. Non-ocular continuous variables were tested using the Mann–Whitney U test. Continuous ocular variables were analyzed using analysis of variances (ANOVA). The association between ocular continuous variables was investigated with the use of Pearson’s correlation. We nested the ANOVA for this factor in order to eliminate bilaterality as a confounding variable. The correlation between the tear parameters was also tested. Values of <0.05 were considered significant. Statistical analyses were performed using Statistica 13.3 (Tibco, Palo Alto, CA, USA).

## 4. Results

There were 72 COVID-19 eyes and 50 control eyes out of the 61 subjects included in the analysis. A majority of post-COVID-19 patients were middle-aged (52.29 ± 13.09) women, with only nine (28%) being male. All patients were white. Age was not significantly different between the groups (50.43 ± 10.31 vs. 51.05 ± 10.43 years in COVID and control, respectively) *p* = 0.78. Most post-COVID-19 patients had hypertension at 33%, whereas hypothyroidism was diagnosed in 11%, while hyperthyroidism was present in 8%. Diabetes type II and asthma were diagnosed in 6% of the patients. Among the COVID-19 group, 36% of patients were hospitalized, while 38% had oxygen supplementation.

**Figure 1:**
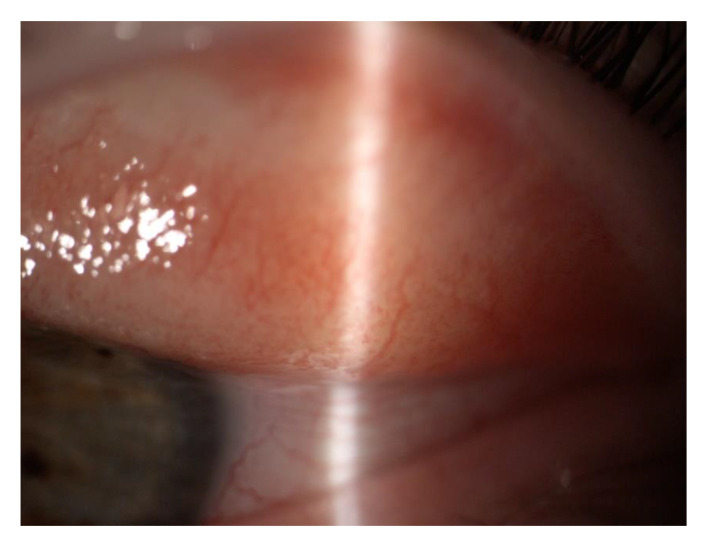
Follicle formation on the conjunctiva of the eyelid accompanying COVID-19 infection.

**Figure 2:**
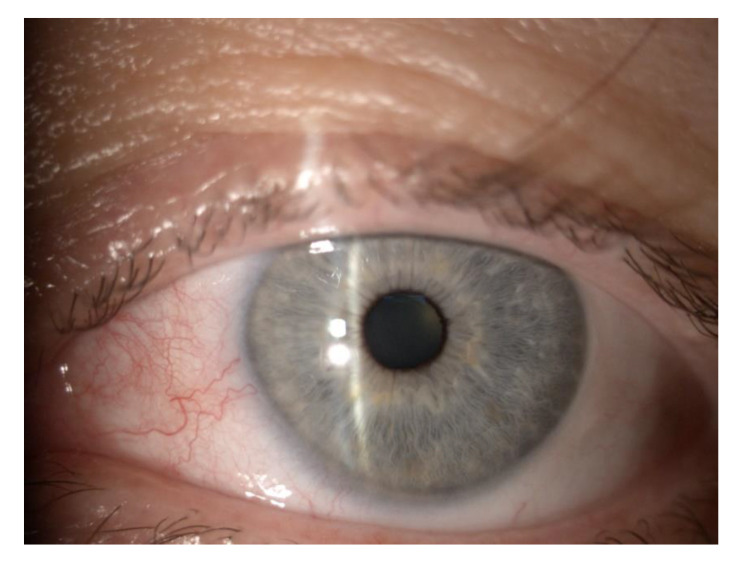
Eye of the patient with episcleritis with onset during COVID-19 infection.

Eye itching and burning (19%) were the most common symptoms of COVID-19 infection, followed by a subjective decrease in vision (17%). In addition, conjunctivitis (Figure 1) and eye pain were present in 11%, whereas 6% of the patients had episcleritis (Figure 2). The mean time from initial infection was 6.5 ± 3.9 (range 1–24 weeks).

Meniscus height was not found to have significantly changed between the COVID-19 (0.34 ± 0.13 mm) group and the control (0.33 ±0.12 mm, *p* = 0.88) group. Neither was this the case in regard to NIKBUT-1 (*p* = 0.88; 7.22 ± 4.60 s and 6.91 ± 4.45 s) results. Further, the NIKBUT average was also non-significant (*p* = 0.91) in the COVID-19 (12.30 ± 5.86 s) group, as well as in the control (11.77 ± 4.97 s) group. Furthermore, IOP did not show differences between the COVID-19 (14.56 ± 2.10 mmHg) group nor in the control (14.11 ± 1.96 mmHg (*p* = 0.17)) group (Table 1).

The Schirmer test was 20.22 ± 7.92 mm in the COVID-19 group and 20.02 ± 7.17 mm in the control (*p* = 0.18) group. Further, Tosm was also not significantly different between the COVID (294.42 ± 54.51 mOsm/dL) group and the control (299.33 ± 5.65 mOsm/dL, *p* = 0.16) group.

No significant violation of CCT was found (549.15 ± 28.98 vs. 539.21vs. 29.08 µm, *p* = 0.06). Neither was the endothelial cell density (2516.64 ± 287.61 vs. 2454.21 ± 498.60 cells/mm^2^) impacted by COVID-19 for *p* = 0.07 and *p* = 0.40, respectively. 

In the COVID-19 groups, the correlation test analysis showed that the NIKBUT average was significantly correlated with NIKBUT-1 (r = 0.73, *p* < 0.01), Tosm (r = −0.53, *p* = 0.03), and meniscus height (r = −0.69, *p* < 0.01) (Table 2). BCVA was significantly correlated with age (r = 0.54, *p* = 0.03) and endothelial cell density (r = −0.84, *p* < 0.01). Additionally, NIKBUT-1 correlated with Tosm (r = −0.67, *p* < 0.01) (Table 2). Similarly, the control NIKBUT-1 tests significantly correlated with Tosm (r = 0.37, *p* = 0.04) and NIKBUT-average (r = 0.82, *p* < 0.01) tests (Table 3). Further NIKBUT-1 was correlated with CCT (r = −0.40, *p* = 0.02) and endothelial cell density (r = 0.37, *p* = 0.04). BCVA correlated with endothelium density only (r = 0.44, *p* = 0.02). Interestingly no correlation was found between the Schirmer test and the tear film parameters in either group (Table 2 and Table 3).

## 5. Discussion

To summarize, this is a retrospective study of the ocular surface symptoms caused by COVID-19. We did not find any significant changes in tear film parameters between the study and the control group. Further, the corneal pachymetry of the COVID-19 patients was not different, nor was the corneal endothelial cell density. Despite there being as many as 591 million coronavirus cases, the COVID-19 disease is still being investigated in order to fully understand its impact on different organs and general health. As the virus has spread all over the world and recurs periodically, it is crucial to fully understand its pathology. The main symptoms are connected mainly to the respiratory tract, though eye symptoms have been also reported. A systematic review and meta-analysis performed by K. Aggrawal et al. revealed that the majority of ophthalmic features reported with COVID-19 were ocular pain, redness, and follicular conjunctivitis [9].

In the study performed by Hong et al. with 56 patients enrolled, detailed ocular symptoms, including eye itching, sore eye, dry eye, and floaters were investigated. In the study, ocular symptoms occurred in 15 (27%) patients and 6 (11%) had prodromal ocular symptoms before disease onset. A cross-sectional study of 534 cases with COVID-19 in Wuhan found the following four of the most common symptoms: conjunctival congestion (4.68%), dry eye (20.97%), blurred vision (12.73%), and foreign body sensation (11.80%) [5]. These results are similar to those described in our study with eye itching and burning (19%) being the most common symptoms of COVID-19 infection, followed by a subjective decrease in vision (17%), conjunctivitis, and eye pain present in 11% and 6% with episcleritis.

Nevertheless, it is unclear whether all these symptoms were caused by COVID-19 infection. The incidence of dry eye disease is high and ocular disorders such as dry eyes, itching, and foreign body sensation are very common in the general population [9,10]. Moreover, the outbreak of the coronavirus pandemic forced the wearing of protection masks, which is a risk factor for dry eye disease. Air convection does influence ocular surface complications and mask-associated dry eye (MADE), which can be caused by poor fitting masks and air leaks, as described in various studies [11,12,13,14]. Dehydration caused by masks has also been proposed as a mechanism for meibomian oil hardening and chalazion formation. In the study performed by R. Silkiss et al., it was also noted that the increased incidence of chalazion may be associated with the widespread adoption of facial mask wear [15].

Considering these reports, it is hard to distinguish if the worsening of dry eye symptoms is caused by coronavirus infection or by face mask wearing instead. Due to safety reasons, the elimination of wearing facial coverings in order to examine the influence of only COVID-19 infection on the ocular surface is impossible. This is because the knowledge gained by doing this would not outweigh the importance of the protective benefits of wearing masks during the pandemic.

With the discovery of the long-COVID conception, described as a multi-organ disease including fatigue, dyspnea, chest pain, cognitive disturbances, and arthralgia after acute COVID- 19, interest in the persistent consequences of the disease, including ocular surface, increased. It is believed that further studies of sequelae after recovery from the acute phase of the disease are necessary in order to develop an evidence-based multidisciplinary approach for the caring of and treating of these patients [16]. In our study, the objective measurements of ocular surface conditions, performed after the acute phase of COVID-19, such as NIKBUT, tear osmolarity, meniscus height, and the Schirmer’s test have not shown any significant differences in the statistical analyses between the post-COVID and control groups. Nevertheless, we have not collected data regarding systemic drugs, which could have influenced some ocular surface parameters. These results are the opposite of what was found in the study performed by K. Wan et al., where the severity of meibomian gland dysfunction and ocular surface staining score was higher in post-COVID-19 than in healthy eyes, when assessed 52.23 ± 16.12 days after infection [6]. Additionally, Gambini G. et al. demonstrated that the post-COVID-19 group showed a higher number of patients with a simultaneous impairment as per their OSDI score and TBUT, along with the Schirmer test results and tear osmolarity significant differences from the control group [17]. These inconsistencies in results between the studies may result from other factors having an impact on tear film parameters, such as working with air conditioners, spending a considerable time in front of computers, and the wearing of face masks for long periods of time. Therefore, further studies are required in order to eliminate these contributing factors.

Furthermore, we showed that tear osmolarity and other tear film parameters can be influenced by physical exercise (the patients were rested before performing the measurements) [18,19], other studies do not provide information about such procedures that potentially could have altered the results. 

Tosm correlated with the NIKBUT positively in the controls and negatively in the study groups. NIKBUT is a measure of tear film instability while Tosm is a measure of tear film concentration as hyperosmolarity was found to be one of the main factors in dry eye pathophysiology. Other authors did not find any correlations between the two [20,21]. As TBUT mainly depends on the lipid layer, the correlation between TBUT and Tosm may not be present in every patient [22]. Except for TBUT and Tosm, we did not find any categories in which we would find a correlation in both groups. We did not find any correlation between the Schirmer test and tear film parameters. This should not come as a surprise as there are two types of dry eye syndrome, one with a decrease in tear production, and one with normal tear production [22]. Our participants had a normal Schirmer test of around 20 mm as well. 

Although respiratory droplets are the main transmission route, the possibility of infection through the ocular surface has also been investigated [23,24,25,26]. Indeed, some studies have demonstrated viral RNA detection in ocular swabs with PCR tests, implicating that tears may be a route of transmission [27,28]. However, some researchers have not revealed any RNA from the studied cohort in conjunctival swabs [29,30]. It also may lead to doubt whether the eye disorders during COVID-19 are caused by the infection of ocular tissues, or are they are a spectrum of the flu-related symptoms accompanying another viral illness [31,32].

## 6. Conclusions

To conclude, the exact incidence and mechanism of ocular findings, especially dry eye disease, in correlation with SARS-CoV-2 requires further research. The inconsistency of the prevalence of dry eye symptoms in COVID-19 across the studies could be confounded by other unobserved factors, especially wearing facemasks during the COVID-19 pandemic.

## Figures and Tables

**Table 1:** Clinical and demographic characteristics of enrolled patients.

COVID-19				
	Mean	Minimum	Maximum	Std. Dev.
Weeks from COVID	6.45	1.00	24.00	3.93
Age (years)	52.29	28.00	84.00	13.09
BCVA	0.93	0.20	1.00	0.20
BCVA logMAR	−0.03	−0.70	0.00	−0.71
IOP (mmHg)	14.56	10.00	19.00	2.10
CCT	549.15	493.00	639.00	28.98
Tosm	294.42	0.00	399.00	54.51
NIKBUT-1	7.22	1.59	20.70	4.60
NIKBUT average	12.30	3.66	24.09	5.86
Schirmer	20.22	8.00	35.00	7.92
Endothelium density	2516.64	1813.00	3360.00	287.61
CCT	549.15	493.00	639.00	28.98
Meniscus height	0.34	0.09	0.70	0.13
**Control**				
Age controls	51.05	31.00	73.00	10.43
IOP (mmHg)	14.11	10.00	18.00	1.96
BCVA	0.94	0.50	1.00	0.12
BCVA logMAR	−0.03	−0.30	0.00	−0.91
CCT	539.21	493.00	599.00	29.08
Tosm	299.33	289.00	309.00	5.65
NIKBUT-1	6.91	1.47	18.93	4.45
NIKBUT average	11.77	1.90	22.05	4.97
Schirmer	20.02	6.00	36.00	7.17
Endothelium density	2454.21	102.00	3100.00	498.60
CCT	539.21	493.00	599.00	29.08
Meniscus height	0.33	0.17	0.62	0.12

**Table 2:** Correlations among the COVID-19 patients’ group, *p* < 0.05, are marked red.

	NIKBUT-1	NIKBUT-Average	Schirmer	Tosm	Endothelium Density	Age	BCVA logMAR	CCT	Meniscus
NIKBUT- 1		0.73	−0.05	−0.67	0.01	0.27	−0.02	0.13	−0.46
NIKBUT-average	0.73		0.32	−0.53	0.22	0.31	0.12	0.18	−0.69
Schirmer	−0.05	0.32		0.42	−0.07	−0.06	0.04	0.32	−0.12
Tosm	−0.67	−0.53	0.42		−0.43	−0.27	−0.34	0.18	0.47
Endothelium density	0.01	0.22	−0.07	−0.43		−0.24	0.84	0.08	−0.31
Age	0.27	0.31	−0.06	−0.27	−0.24		−0.54	−0.25	−0.05
BCVA	−0.02	0.12	0.04	−0.34	0.84	−0.54		0.13	−0.31
CCT	0.13	0.18	0.32	0.18	0.08	−0.25	0.13		−0.48
Meniscus	−0.46	−0.69	−0.12	0.47	−0.31	−0.05	−0.31	−0.48	

**Table 3:** Correlations among the control group *p* < 0.05 are marked red.

	NIKBUT-1	NIKBUT Average	Schirmer	Endothelium Density	Tosm	Age	BCVA logMAR	CCT	Meniscus
NIKBUT- 1		0.82	0.09	0.36	0.37	0.08	−0.21	−0.40	−0.09
NIKBUT-average	0.82		0.12	0.15	0.29	0.13	−0.12	−0.45	−0.03
Schirmer	0.09	0.12		−0.30	0.18	−0.12	−0.09	0.00	−0.12
Endothelium density	0.36	0.15	−0.30		0.11	0.08	−0.40	−0.06	0.21
Tosm	0.37	0.29	0.18	0.11		0.09	−0.17	0.33	0.30
Age	0.08	0.13	−0.12	0.08	0.09		−0.02	−0.22	0.15
BCVA	−0.21	−0.12	−0.09	−0.40	−0.17	−0.02		0.02	−0.20
CCT	−0.40	−0.45	0.00	−0.06	0.33	−0.22	0.02		0.23
Meniscus	−0.09	−0.03	−0.12	0.21	0.30	0.15	−0.20	0.23	

## Data Availability

Data are available on request due to restrictions, i.e., privacy or ethical concerns.

## Abbreviations

PCR: polymerase chain reaction; IOP: intraocular pressure; NIKBUT: non-invasive keratograph break-up time; Tosm: tear osmolarity; CCT: central corneal thickness; and BCVA: best corrected visual acuity.

## References

[1] Szkodny D., Wylęgała E., Sujka-Franczak P., Chlasta-Twardzik E., Fiolka R., Tomczyk T., Wylęgała A. (2021). Retinal oct findings in patients after covid infection. J. Clin. Med..

[2] Lauermann P., Storch M., Weig M., Tampe B., Winkler M., Hoerauf H., Feltgen N., Hakroush S. (2020). There is no intraocular affection on a SARS-CoV-2-Infected ocular surface. Am. J. Ophthalmol. Case Rep..

[3] Sen M., Honavar S.G., Sharma N., Sachdev M.S. (2021). COVID-19 and Eye: A Review of Ophthalmic Manifestations of COVID-19. Indian J. Ophthalmol..

[4] Roehrich H., Yuan C., Hou J.H. (2020). Immunohistochemical Study of SARS-CoV-2 Viral Entry Factors in the Cornea and Ocular Surface. Cornea.

[5] Chen X., Yu H., Mei T., Chen B., Chen L., Li S., Zhang X., Sun X. (2021). SARS-CoV-2 on the ocular surface: Is it truly a novel transmission route?. Br. J. Ophthalmol..

[6] Wan K.H., Lui G.C.Y., Poon K.C.F., Ng S.S.S., Young A.L., Hui D.S.C., Tham C.C.Y., Chan P.K.S., Pang C.P., Chong K.K.L. (2022). Ocular surface disturbance in patients after acute COVID-19. Clin. Exp. Ophthalmol..

[7] Ioannou G.N., Baraff A., Fox A., Shahoumian T., Hickok A., Hare A.M.O. (2022). Rates and Factors Associated with Documentation of Diagnostic Codes for Long COVID in the National Veterans Affairs Health Care System. JAMA Netw. Open.

[8] Aiyegbusi O.L., Hughes S.E., Turner G., Rivera S.C., McMullan C., Chandan J.S., Haroon S., Price G., Davies E.H., Nirantharakumar K. (2021). Symptoms, complications and management of long COVID: A review. J. R. Soc. Med..

[9] Aggarwal K., Agarwal A., Jaiswal N., Dahiya N., Ahuja A., Mahajan S., Tong L., Duggal M., Singh M., Agrawal R. (2020). Ocular surface manifestations of coronavirus disease 2019 (COVID-19): A systematic review and meta-analysis. PLoS ONE.

[10] Smith J.A., Albenz J., Begley C., Caffery B., Nichols K., Schaumberg D. (2007). The epidemiology of dry eye disease: Report of the epidemiology subcommittee of the international Dry Eye WorkShop (2007). Ocul. Surf..

[11] Shalaby H.S., Eldesouky M.E.E.S. (2022). Effect of facemasks on the tear film during the COVID-19 pandemic. Eur. J. Ophthalmol..

[12] Dag U., Çaglayan M., Öncül H., Vardar S., Alaus M.F. (2022). Mask-associated Dry Eye Syndrome in Healthcare Professionals as a New Complication Caused by the Prolonged Use of Masks during Covid-19 Pandemic Period. Ophthalmic Epidemiol..

[13] Krolo I., Blazeka M., Merdzo I., Vrtar I., Sabol I., Petric-Vickovic I. (2021). Mask-Associated Dry Eye During COVID-19 Pandemic-How Face Masks Contribute to Dry Eye Disease Symptoms. Med. Arch..

[14] Esen Baris M., Guven Yilmaz S., Palamar M. (2022). Impact of prolonged face mask wearing on tear break-up time and dry eye symptoms in health care professionals. Int. Ophthalmol..

[15] Silkiss R.Z., Paap M.K., Ugradar S. (2021). Increased incidence of chalazion associated with face mask wear during the COVID-19 pandemic. Am. J. Ophthalmol. Case Rep..

[16] Nalbandian A., Sehgal K., Gupta A., Madhavan M.V., McGroder C., Stevens J.S., Cook J.R., Nordvig A.S., Shalev D., Sehrawat T.S. (2021). Post-acute COVID-19 syndrome. Nat. Med..

[17] Gambini G., Savastano M.C., Savastano A., De Vico U., Crincoli E., Cozzupoli G.M., Culiersi C., Rizzo S. (2021). Ocular Surface Impairment After Coronavirus Disease 2019: A Cohort Study. Cornea.

[18] Wylęgała A., Sędziak-Marcinek B., Pilch J., Wylęgała E. (2019). Alteration of Blinking and Sex Differences During Physical Exercise Affect Tear Osmolarity. J. Hum. Kinet..

[19] Lee J.H., Kim C.H., Choe C.M., Choi T.H. (2020). Correlation Analysis between Ocular Surface Parameters with Subjective Symptom Severity in Dry Eye Disease. Korean J. Ophthalmol..

[20] Berchicci L., Aragona E., Arrigo A., Marchese A., Miserocchi E., Bandello F., Modorati G. (2021). Conjunctival Matrix Metalloproteinase-9 Clinical Assessment in Early Ocular Graft versus Host Disease. J. Ophthalmol..

[21] Tsubota K. (2018). Short Tear Film Breakup Time-Type Dry Eye. Investig. Ophthalmol. Vis. Sci..

[22] Matos A.G., Sarquis I.C., Santos A.A.N., Cabral L.P. (2021). Covid-19: Risk of ocular transmission in health care professionals. Rev. Bras. Med. Trab..

[23] Petronio Petronio G., Di Marco R., Costagliola C. (2021). Do Ocular Fluids Represent a Transmission Route of SARS-CoV-2 Infection?. Front. Med..

[24] Willcox M.D.P., Walsh K., Nichols J.J., Morgan P.B., Jones L.W. (2020). The ocular surface, coronaviruses and COVID-19. Clin. Exp. Optom..

[25] Chen Z., Yuan G., Duan F., Wu K. (2020). Ocular Involvement in Coronavirus Disease 2019: Up-to-Date Information on Its Manifestation, Testing, Transmission, and Prevention. Front. Med..

[26] Li M., Yang Y., He T., Wei R., Shen Y., Qi T., Han T. (2021). Detection of SARS-CoV-2 in the ocular surface in different phases of COVID-19 patients in Shanghai, China. Ann. Transl. Med..

[27] Long Y., Wang X.W., Tong Q., Xia J.H., Shen Y. (2020). Investigation of dry eye symptoms of medical staffs working in hospital during 2019 novel coronavirus outbreak. Medicine.

[28] Seah I.Y.J., Anderson D.E., Kang A.E.Z., Wang L., Rao P., Young B.E., Lye D.C., Agrawal R. (2020). Assessing Viral Shedding and Infectivity of Tears in Coronavirus Disease 2019 (COVID-19) Patients. Ophthalmology.

[29] Kocamış Ö., Örnek K., Aşıkgarip N., Hızmalı L., Sezgin F.M., Şahin Y. (2021). Evaluation of Nasopharyngeal and Conjunctival Swab Samples of Hospitalised Patients with Confirmed COVID-19. Ocul. Immunol. Inflamm..

[30] Creager H.M., Kumar A., Zeng H., Maines T.R., Tumpey T.M., Belser J.A. (2018). Infection and Replication of Influenza Virus at the Ocular Surface. J. Virol..

[31] Belser J.A., Lash R.R., Garg S., Tumpey T.M., Maines T.R. (2018). The eyes have it: Influenza virus infection beyond the respiratory tract. Lancet Infect Dis..

[32] Abdul-Kadir M.A., Lim L.T. (2020). Human coronaviruses: Ophthalmic manifestations. BMJ Open Ophthalmol..

